# Study on abrasive water jet machining of compacted graphite iron on Taguchi method

**DOI:** 10.1038/s41598-026-57516-z

**Published:** 2026-06-11

**Authors:** Kenan Kütükde

**Affiliations:** Department of Mechanical Program, Kafkas University, Kars, 36100 Turkey

**Keywords:** Compact graphite iron, Abrasive water jet machining, Taguchi, Surface roughness, Kerf width, Kerf taper angle, Engineering, Environmental sciences, Materials science

## Abstract

In this study, the influence of abrasive flow rate (AFR), stand-off distance (SOD), and traverse speed (TS) on surface roughness (SR), kerf width (KW), and kerf taper angle (KTA) during the abrasive water jet machining (AWJM) of 10 mm thick compacted graphite iron (CGI) was investigated. A full-factorial Taguchi L27 orthogonal array was employed to optimize the response parameters in terms of the SR, KW, and KTA. Analysis of variance (ANOVA) was performed to evaluate the statistical significance and contribution ratios of each parameter. The results revealed that AFR was the most dominant factor affecting SR and KTA, whereas TS had the greatest influence on KW. In contrast, SOD had only a minor impact on all the response variables. The optimized machining conditions for CGI were determined as 325 g/min AFR, 1.5 mm SOD, and 20 mm/min TS for the minimum SR. The findings highlight that the AWJM process can effectively machine CGI with high precision and minimal thermal damage, offering a viable alternative to conventional methods for difficult-to-machine materials.

## Introduction

CGI materials, characterized by a carbon content ranging from 3.5% to 3.8%, exhibit a tensile strength approximately 70% higher and an elastic modulus 35% greater than that of gray cast iron. These advantages have made CGI increasingly desirable for producing diesel engine components and other demanding applications in recent years^1–4^. The microstructure of CGI, composed of worm-shaped, short, and thick compressed graphite particles, enhances the bonding between the graphite and iron matrix, significantly improving the fatigue strength up to twice that of gray cast iron^2^. However, although graphite contributes positively to the mechanical properties, it also reduces the thermal conductivity at room temperature by up to 25% compared with gray cast iron^3^. In contrast, the increased sphericity and proportion of compacted graphite in CGI materials pose challenges to their castability and machinability using conventional methods^1,4^.

AWJM has emerged as an effective unconventional manufacturing method, characterized by low cutting forces and minimal cutting temperatures^5–8^. Moreover, the preservation of the mechanical and chemical properties of the machined surface after processing distinguishes AWJM as a superior machining method. This unconventional manufacturing method has been extensively utilized for the precise cutting of a diverse array of materials, including composites, metals, non-metallic plastics, glass, concrete, marble, and wood, etc.^6,7,9–12^. In this method, response values such as the material removal rate (MRR), SR, KW, and KTA are determined by machining parameters such as the type of material, material thickness, type of abrasive, size of abrasive, abrasive-water mixture ratio, water jet pressure (WJP), SOD, and TS. The effects of machining parameters on different types of materials have been the subject of research.

For this purpose, researchers have studied the effects of machining parameters in AWJM on different types of ferrous materials. It has been reported that the effect of SOD on the SR in AISI 316 L steels is minimal, whereas the TS is the most important parameter^7^. In addition, the surface quality improved with increasing WJP. In AISI 316 L steels, with decreasing cutting speed, the SR value decreased, and the width of the primary zone without machining marks increased^8^. It was concluded that the main factor affecting the SR and KTA in mild steel sheets was the TS, while the other effective parameters were the SOD and AFR, respectively^9^. It was concluded that the TS is effective on the MRR and SR in AISI D3 steels; an increase in the TS improves the MRR, and a decrease in the TS improves the surface quality^10^. In Duplex 2205 steels, the effects of machining parameters on SR were SOD with 39.16%, AFR with 16.24%, and TS with 5.27%, while the effects on KTA were TS with 54.85%, SOD with 20.91%, and AFR with 19.27%, respectively^11^. In EN31 steels, TS was effective on SR, whereas other machining parameters had no significant effect^12^. In addition, the SR increased with an increase in TS and decreased with an increase in pressure. In another study conducted on EN31 steel, WJP was identified as the dominant factor, and when combined with high TS and maximum AFR, superior surface quality was achieved^13^. In 316 Stainless Steel, SOD was the most influential parameter, followed by TS, then AFR^14^. A higher TS generally enhances the surface quality, whereas an excessive AFR tends to deteriorate it owing to the particle crowding effects. It has been reported that TS and AFR are the most important factors affecting MRR and KTA in stainless steels^15^. With increases in the AFR and TS, both the MRR and KTA increased. In AISI 304 steels, TS was the most important factor affecting the MRR and SR, whereas SOD was the fourth most important factor^16^. The most important parameter for EN24 steels of different thicknesses is the TS, followed by the WJP^17^. Deterioration of the SR was observed as the thickness of the machined material increased. The effects of machining parameters on the SR in AISI 1045 steels were found to be pressure with 56.46% and cutting feed with 28.99%^18^. The most effective machining parameter for MRR was the cutting feed, with a value of 87.33%. SR increased with increasing pressure, whereas MRR increased with decreasing pressure and SOD. In AISI 304 L steels, the SR decreased with increasing WJP and AFR, whereas the MRR increased^19^. Increasing the TS and material thickness increased the SR. For mild steel materials, the most important parameter affecting the SR was the TS, whereas the effects of the WJP and SOD on the SR were insignificant^20^. In Rolled Homogeneous Armor (RHA) materials, the most important factor is TS, followed by SOD^21^. In AISI 1018 steels, the feed rate was found to be the most important parameter affecting the SR and KTA^22^. Both the SR and KTA decreased with decreasing feed rate. In 304 stainless steels, the most effective parameters for MRR were the water pressure and feed rate^23^. An increase in the feed rate and a decrease in the AFR resulted in a minimum kerf top width. In AISI 1018 steels, an increase in the AFR decreased both the SR and KTA, whereas an increase in the SOD and TS increased both the SR and KTA^24^. In Austempered Ductile Iron materials, the effects of AFR, SOD, and nozzle speed on SR, KTA, and MRR were minimal, whereas the most important parameter was WJP^25^. The increase in WJP increased the MRR while decreasing the SR and KTA values, respectively. In Fe-Based Shape Memory Alloy materials, WJP was the most influential parameter, followed by TS, whereas SOD and AFR showed low influence^26^. Surface quality improved with decreasing SOD and increasing TS. In high-thickness St37 steel plates, TS was the most important factor affecting the SR, waviness, and KTA, followed by AFR^27^. Although water pressure was effective on waviness and KTA, it did not significantly affect SR. In SS304 stainless steel, the contributions of WJP and TS to the chip depth were greater than that of AFR^28^. The penetration depth increased with increasing WJP and AFR and decreased with increasing TS. A high-carbon steel plate (61 mm thick) and a high-strength concrete cube (150 mm thick) were cut using seven different abrasive materials. Choromite and zirconium grains showed poor cutting performance, whereas unsized Australian and sized Ukrainian garnets showed similar cutting performances^29^.

CGI materials have increasingly replaced gray cast iron in the production of next-generation diesel engines owing to their superior tensile strength, adequate thermal conductivity, and lightweight. Diesel engines utilizing CGI materials are particularly advantageous because they enhance fuel efficiency and contribute to environmental sustainability by reducing carbon emissions. Despite these significant advantages, the machinability of CGI materials with AWJM, which challenges conventional machining methods, has not been sufficiently researched. Hence, this study aimed to determine the optimal machining parameters for the AWJM of CGI using the Taguchi L27 orthogonal design and signal-to-noise (S/N) ratio analysis. The effects of AFR, SOD, and TS on SR, KW, and KTA were evaluated using ANOVA to quantify their individual and interactive contributions to the observed effects. The results are expected to provide valuable insights for improving the AWJM performance when machining hard-to-machine CGI materials.

## Experimental process

**Fig. 1:**
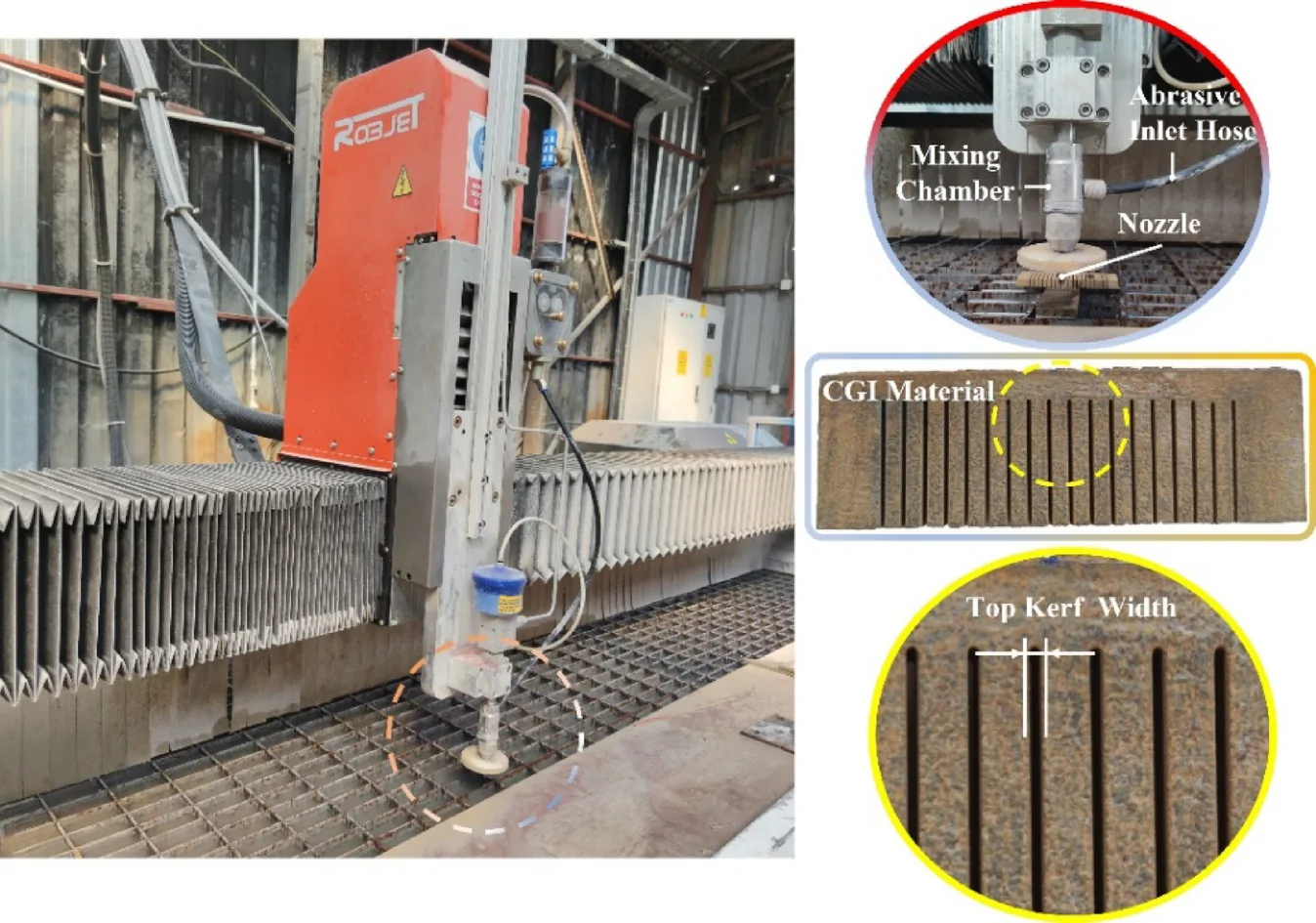
Overview of the Robjet CUTJET-W3-4020 machine.

**Fig. 2:**
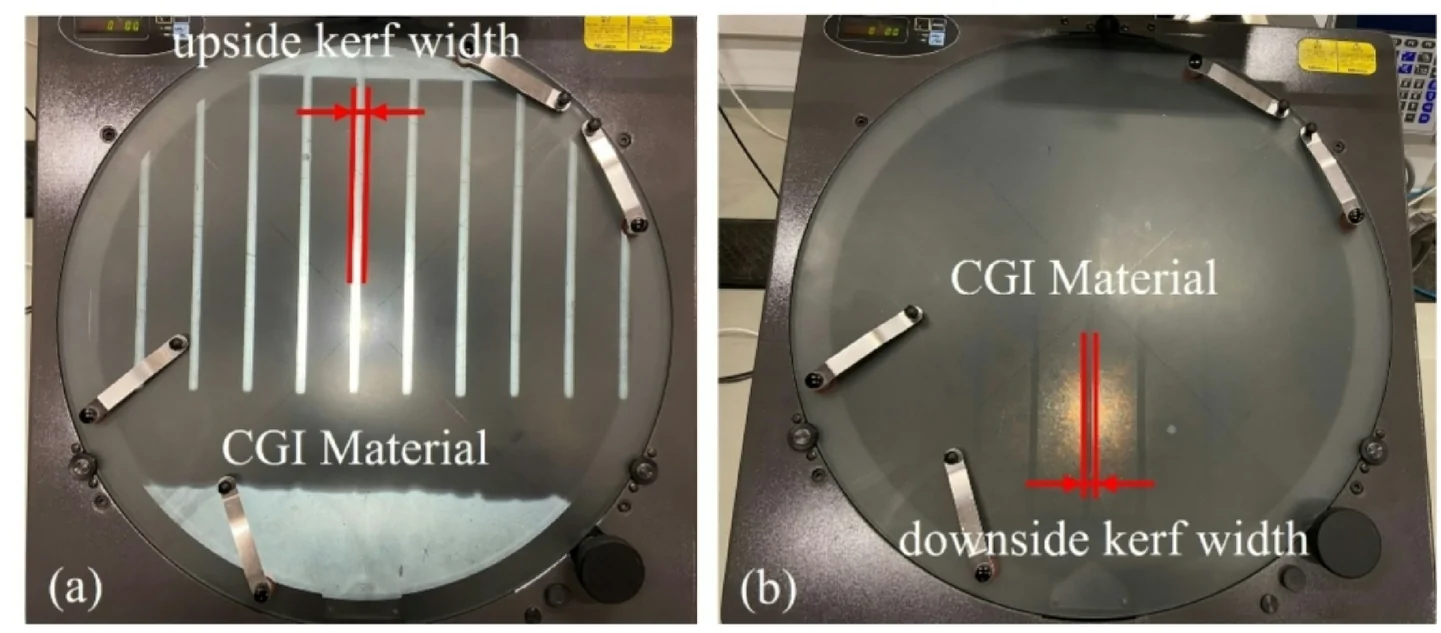
KW measurement on Mititoyo P-V 5000 profile projector **(a)** upside **(b)** downside.

An experimental study was conducted on CGI plates with dimensions of 125$\:\times\:$40$\:\times\:$10 mm (Fig. 1(a)). The chemical composition and mechanical properties of the material are listed in Tables 1 and 2, respectively. The experiments were performed using an Australian garnet abrasive with a mesh size of 80 μm. The experiments were conducted using an Robjet CUTJET-W3-4020 water jet cutting system (Fig. 1(b)), the technical specifications of which are summarized in Table 3. During the experiments, a 0.01” sapphire orifice and a 0.281$\:\times\:$0.03$\:\times\:$3” Roctech Accusstream carbide focusing tube were used. The technological parameters used in the experiments are presented in Table 4. To investigate the impact of the AWJM parameters on the CGI material, a Taguchi L27 orthogonal array experimental design was used. For this purpose, three different levels were assigned to each of the three process parameters (AFR, SOD, and TS) (Table 5). A Mititoyo PV-5000 profile projector with 20$\:\times\:$ optical magnification was used the precise measurement of the KW. The measurement of the entrance width of the grooves formed as a result of the cutting process is shown in Fig. 2(a), and the measurement of the exit width is shown in Fig. 2(b). The SR values (Ra) were evaluated in accordance with the ISO 21920-2 (formerly 1997) standard using a Mitutoyo Surftest SJ-210 device on the upper, middle, and lower regions of the material. The average of the upper and middle regions was considered, whereas the lower region was excluded because of the energy loss caused by water jet.

**Table 1:** Chemical composition of CGI (% ratio).

C	Si	Mn	*P*	S	Cr	Ni	Mo
3.82	1.804	0.337	0.031	0.015	0.074	0.013	0.002
Cu	Mg	Sn	Ti	Al	Zn	Bi	Fe
0.879	0.014	0.092	0.0203	0.008	0.082	0.007	Rest

**Table 2:** Mechanical properties of CGI.

Max. Tensile Strength (MPa)	% 0.2 Yield Strength (MPa)	Elongation (%)	Hardness of Vickers (HV)	Impact Strength (Joule)
502.7	284.3	1.8	280	8.6

**Table 3:** Properties of the Robjet CUTJET-W3-4020 machine.

Property	Defaults
CNC Control Unit	Fagor / Beckhoff
Screen Dimensions	279.4 mm/533.4 mm (11” / 21”)
Maximum Working Area	4000 × 2000 × 200 mm
Maximum Speed in x and y axis	60 m/min
Maximum Acceleration in x and y axis	10 m/s^2^
Maximum Traverse Speed	2 m/min
Positional Accuracy	± 0.05 mm
Repeatability	± 0.03 mm
Abrasive Flow Rate	406 g/min
Feeding Power	380 V AC 50 Hz 3 Ph

**Table 4:** Fixed parameters used in AWJM.

Technological Parameters	Unit	Value
Material thickness	mm	10
Water Pressure	MPa	300
Abrasive type		Australian mineral garnet
Abrasive particle size	Mesh	80 (0.177 mm)
Orifice dimension	Inch	0.001
Nozzle Dimensions	Inch	0.281 × 0.03 × 3
Impact Angle	degree	90

**Table 5:** Processing Factors and Levels of AWJM.

Factors	Unit		Levels	
		1	2	3
Abrasive flow rate	g/min	244	325	406
Nozzle stand-off distance	mm	1.5	2	2.5
Traverse Speed	mm/min	15	20	25

## Result and discussion

The Taguchi method is widely applied to identify optimum parameter values, offering significant savings in time and cost by requiring fewer experiments compared to conventional approaches. Moreover, it enhances the reliability of results by establishing a robust relationship between process parameters and response variables^30^. In this study, the Taguchi L27 orthogonal array was employed, as it enables a systematic evaluation of multiple processing parameters (e.g., AFR, SOD, and TS) at three distinct levels. This design provides an effective balance between experimental efficiency and robustness, while yielding clear insights into both the individual and interactive effects of these parameters on response variables (e.g., SR, KW, and KTA). According to the Taguchi L27 orthogonal array experimental design, the KW and SR (Ra) values at each factor and level were recorded (Table 6). After measuring the kerf widths, the KTA was calculated using the following Eqs.^6,9,11,18,21,27^:

1 $$\:Kerf\:Taper\:Angle\:\left(KTA\right)={\mathrm{tan}}^{-1}\frac{\left({W}_{t}-{W}_{b}\right)}{2xt}$$

In Eq. (1), $\:{W}_{t}$ = upper KW, $\:{W}_{b}$ = lower KW, and t = CGI material thickness.

In the machining of CGI materials with AWJM, the “smaller-is-better” criterion was adopted for calculating the S/N ratios to achieve the desired minimum output values. This approach provides an effective strategy for optimizing machining conditions, as it promotes smoother surfaces, narrower kerfs, and straighter cuts, which are critical quality indicators in AWJM^31–33^. The following formula was used to calculate the S/N ratios^19,30^:

2 $$\:S/N\:oranı\:\left(\eta\:\right)=-10{\mathrm{log}}_{10}(\frac{1}{n}{\sum\:}_{i=1}^{n}{{y}^{2}}_{ij})$$

In Eq. (2), n is the number of repetitions of the measured characteristic value and Yi is the measured characteristic value (SR, KW, and KTA). The S/N ratios for SR, KW, and KTA were calculated and are listed in Table 7.

**Table 6:** Experimental results of L27 orthogonal array with three factors and three levels.

Test No	Abrasive flow rate (g/min) (A)	Stand-off distance (mm) (B)	Traverse Speed (mm/min) (C)	Average Roughness of Surface (µm)	Kerf Width (Top) (mm)	Kerf Width (Bottom) (mm)	Kerf Taper Angle (°)
1	244	1.50	15	3.782	0.717	0.673	0.12605
2	244	1.50	20	4.689	0.652	0.601	0.14610
3	244	1.50	25	6.071	0.69	0.635	0.15756
4	244	2	15	4.822	0.729	0.691	0.10886
5	244	2	20	4.267	0.698	0.682	0.04584
6	244	2	25	5.145	0.675	0.651	0.06876
7	244	2.5	15	4.041	0.76	0.734	0.07448
8	244	2.5	20	4.352	0.732	0.679	0.15183
9	244	2.5	25	4.302	0.709	0.664	0.12892
10	325	1.50	15	5.269	0.77	0.753	0.04870
11	325	1.50	20	3.583	0.736	0.684	0.14897
12	325	1.50	25	3.680	0.682	0.673	0.02578
13	325	2	15	3.643	0.774	0.742	0.09167
14	325	2	20	3.710	0.745	0.722	0.06589
15	325	2	25	4.515	0.683	0.654	0.08308
16	325	2.5	15	4.680	0.8	0.798	0.00573
17	325	2.5	20	4.534	0.768	0.753	0.04297
18	325	2.5	25	3.979	0.721	0.687	0.09740
19	406	1.50	15	5.033	0.754	0.62	0.38388
20	406	1.50	20	4.771	0.735	0.641	0.26929
21	406	1.50	25	5.724	0.712	0.617	0.27215
22	406	2	15	4.917	0.812	0.724	0.2521
23	406	2	20	6.206	0.786	0.679	0.30653
24	406	2	25	6.015	0.73	0.613	0.33518
25	406	2.5	15	5.898	0.829	0.731	0.28075
26	406	2.5	20	5.635	0.811	0.717	0.26929
27	406	2.5	25	6.397	0.73	0.612	0.33804

For SR, KW, and KTA, the arithmetic means of the S/N ratios for each level were considered. The difference values were obtained by subtracting the minimum S/N ratio from the maximum S/N ratio for each factor^34^. When the resulting difference values were ranked from largest to smallest, the degree of influence of each factor on SR, KW, and KTA was revealed.

**Table 7:** Calculated S/N ratios for SR, KW and KTA.

Test No	SR (µm)	S/*N* ratio for SR	KW (Top) (mm)	S/*N* ratio for KW	KTA (°)	S/*N* ratio for KTA
1	3.782	-11.55	0.717	2.89	0.12605	17.99
2	4.689	-13.42	0.652	3.72	0.14610	16.71
3	6.071	-15.66	0.69	3.22	0.15756	16.05
4	4.822	-13.67	0.729	2.75	0.10886	19.26
5	4.267	-12.60	0.698	3.12	0.04584	26.78
6	5.145	-14.23	0.675	3.41	0.06876	23.25
7	4.041	-12.13	0.76	2.38	0.07448	22.56
8	4.352	-12.77	0.732	2.71	0.15183	16.37
9	4.302	-12.67	0.709	2.99	0.12892	17.79
10	5.269	-14.44	0.77	2.27	0.04870	26.25
11	3.583	-11.09	0.736	2.66	0.14897	16.54
12	3.680	-11.32	0.682	3.32	0.02578	31.77
13	3.643	-11.23	0.774	2.23	0.09167	20.76
14	3.710	-11.39	0.745	2.56	0.06589	23.62
15	4.515	-13.09	0.683	3.31	0.08308	21.61
16	4.680	-13.41	0.8	1.94	0.00573	44.84
17	4.534	-13.13	0.768	2.29	0.04297	27.34
18	3.979	-11.99	0.721	2.84	0.09740	20.23
19	5.033	-14.04	0.754	2.45	0.38388	8.32
20	4.771	-13.57	0.735	2.67	0.26929	11.40
21	5.724	-15.15	0.712	2.95	0.27215	11.30
22	4.917	-13.83	0.812	1.81	0.2521	11.97
23	6.206	-15.86	0.786	2.09	0.30653	10.27
24	6.015	-15.58	0.73	2.73	0.33518	9.49
25	5.898	-15.41	0.829	1.63	0.28075	11.03
26	5.635	-15.02	0.811	1.82	0.26929	11.40
27	6.397	-16.12	0.73	2.73	0.33804	9.42

### Surface Roughness (Ra)

The arithmetic means of the S/N ratios of each level, differences, and degree of influence of the AWJM parameters on SR are presented in Table 8. Accordingly, when the control factors affecting SR were ranked according to their degree of influence, AFR ranked 1st, TS ranked 2nd and SOD ranked 3rd. In this study, contrary to previous studies on steel, it was observed that the most important parameter affecting the SR was the AFR^7,9,24^. This can be attributed to the unique microstructure and hardness of CGI, which alter the dynamics of the abrasive-surface interaction. The optimum machining parameters for SR were the levels at which each factor had the highest S/N ratio. The largest S/N ratio of each factor (marked with *) is shown in Table 8. Thus, the optimum conditions for SR were determined. The optimal parameter combination for minimizing the SR was an AFR of 325 g/min, SOD of 1.5 mm, and TS of 20 mm/min.

**Table 8:** Average S/N ratios of AWJM parameters for SR.

Levels	A	B	C
1	-13.19	-13.36*	-13.30
2	-12.34*	-13.50	-13.20*
3	-14.95	-13.63	-13.98
Difference	2.61	0.27	0.78
Rank	1	3	2

* = Optimal Level.

**Fig. 3:**
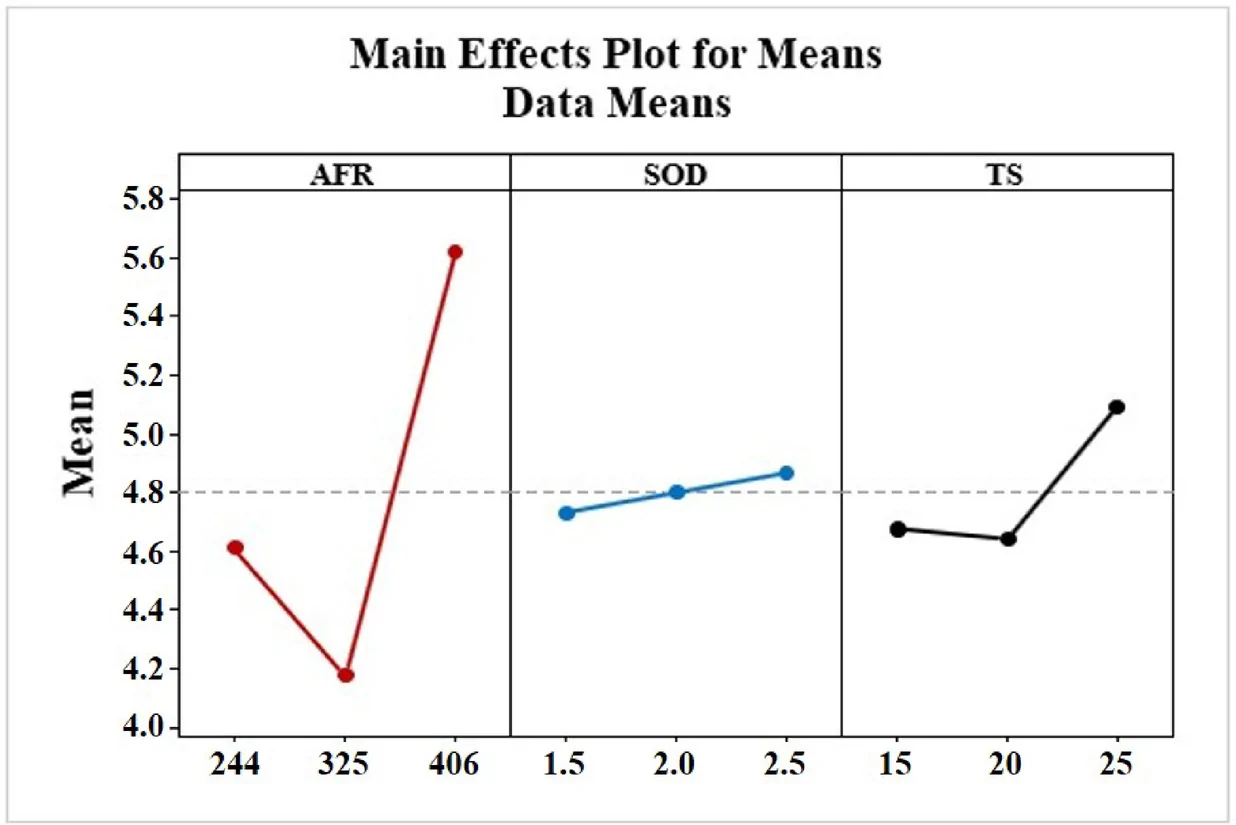
Main effect of AWJM parameters for SR.

The effect graphs of each factor on SR according to the average S/N ratio are shown in Fig. 3. When the graph is examined, it is observed that the SR value decreases while the AFR increases from 244 to 325 g/min, and the SR value increases while the AFR increases from 325 to 406 g/min. SR increased with increasing SOD. The SR value increases with an increase in SOD value^24,26^. The SR value decreased slightly when the TS value increased from 15 to 20 mm/min and increased when the TS value increased from 20 to 25 mm/min.

ANOVA was carried out at a 5% significance level (95% confidence level) to determine the effects and significance of AWJM parameters and their interactions on SR, with the results presented in Table 9^35^. According to the ANOVA results, the most important factor affecting SR was AFR (51.46%). Other factors affecting SR were the interactions of AFR and SOD (9.68%), AFR and TS (9.64%), and SOD and TS (3.94%), respectively. However, the effects of factors other than AFR and their interactions were statistically insignificant (F-test < F-table). Thus, AFR was confirmed as the dominant parameter affecting SR in the AWJM of CGI.

**Table 9:** ANOVA result for the average roughness of surface (Ra).

Factors	DF	SS	V	F-test	PD
AFR (A)	2	9.9010	4.9505	10.876	51.46
SOD (B)	2	0.0820	0.04099	0.090	0.43
TS (C)	2	1.1391	0.5696	1.251	5.92
A$\:\times\:$B	4	1.8625	0.4656	1.023	9.68
A$\:\times\:$C	4	1.8557	0.4639	1.019	9.64
B$\:\times\:$C	4	0.7589	0.1897	0.417	3.94
Error	8	3.6415	0.4552		18.93
Total	26	19.2407	7.1355		

Model summary *S* = 0,674679; $$\:{R}^{2}$$=81.07%; $$\:{R}_{adj}^{2}$$=38.49%.

DF: Degree of Freedom, SS: Sum of Squares, V: Variance, PD: Percentage of.

Distribution F-Table (0.05;2;8) = 4.46, F-Table (0.05;4;8) = 3.84.

**Fig. 4:**
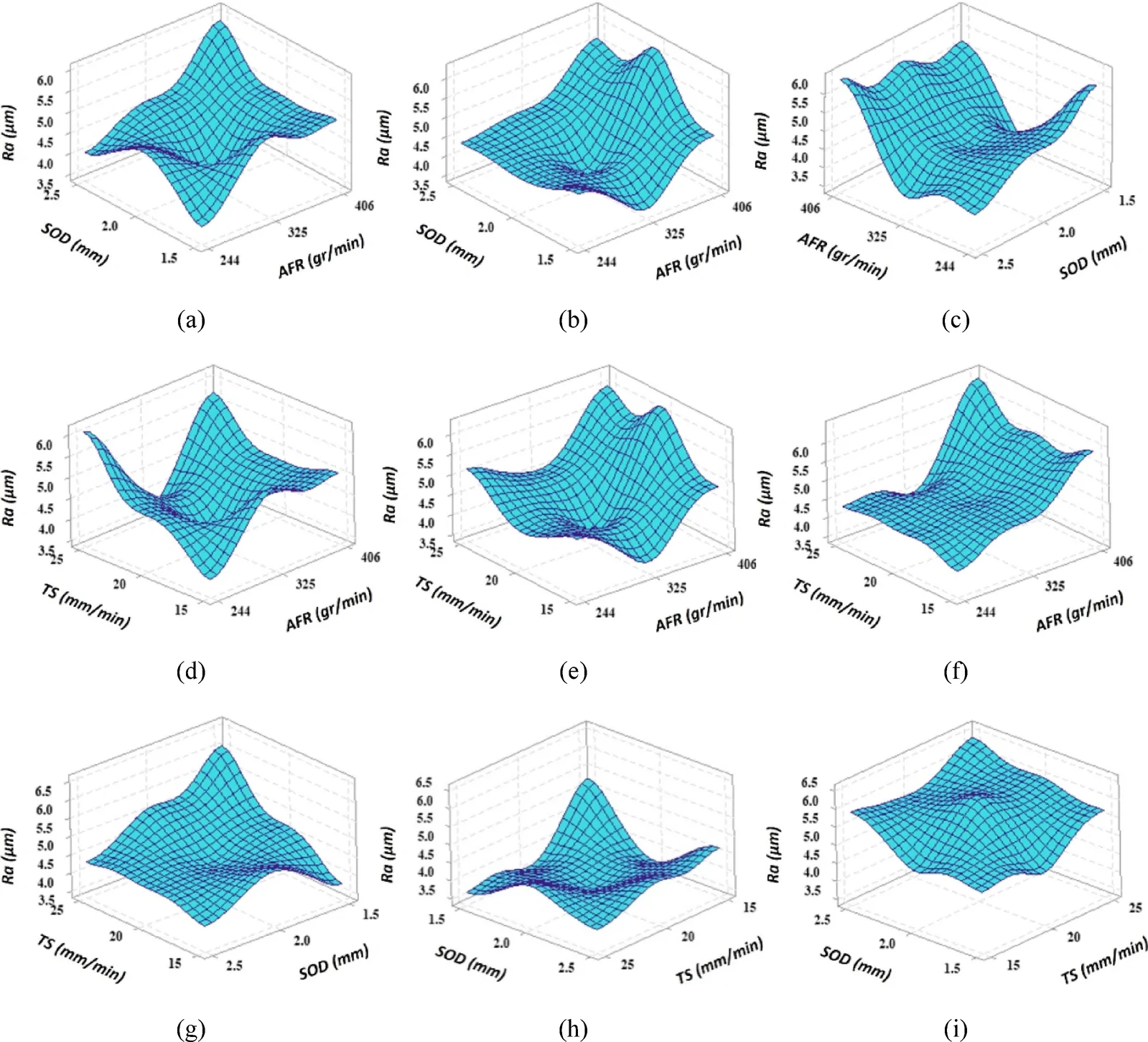
3D surface plot for surface roughness (Ra), **(a)** TS = 15 mm/min **(b)** TS = 20 mm/min **(c)** TS = 25 mm/min **(d)** SOD = 1.5 mm **(e)** SOD = 2 mm **(f)** SOD = 2.5 mm **(g)** AFR = 244 g/min **(h)** AFR = 325 g/min **(i)** AFR = 406 g/min.

Figure 4 shows the effects of the interactions of the AWJM parameters on SR as a 3D surface plot. In Fig. 4(a), TS was fixed at 15 mm/min, in Fig. 4(b) at 20 mm/min, and in Fig. 4(c) at 25 mm/min, and the effect of AFR on SR relative to SOD is shown. When the graphs in Fig. 4 (a) and 4 (b) are examined, it is observed that the SR value increases in response to the increasing values of AFR and SOD. In Fig. 4 (a-c), the SR values decrease at 325 mm/min, which is the middle value of AFR, and at 2 mm, which is the middle value of the SOD.

In Fig. 4 (d), SOD was fixed at 1.5 mm, in Fig. 4 (e) at 2 mm, and in Fig. 4 (f) at 2.5 mm, and the effect of AFR on SR relative to TS is shown. When the graphs in Fig. 4 (d), 4 (e), and 4 (f) are examined, it is observed that the SR value increases in response to the increasing values of AFR and TS. In Fig. 4 (d-f), the SR values decrease at 325 mm/min, which is the middle value of AFR, and at 20 mm/min, which is the middle value of TS.

In Fig. 4 (g), AFR was fixed at 244 mm/min, in Fig. 4 (h) at 325 mm/min, and in Fig. 4 (i) at 406 mm/min, and the effect of TS on SR relative to SOD is shown. When the graphs in Fig. 4 (f-i) are examined, it is observed that the SR value increases in response to increasing values of SOD and TS. In a similar study, it was reported that the SR increased in response to increasing SOD and TS values^9^. In Fig. 4 (g, h), the SR values decrease at 2 mm, which is the middle value of SOD, and at 20 mm/min, which is the middle value of TS. In Fig. 4 (i), the SR value increases at the middle value of SOD and TS. This can be explained by the increase in the AFR.

In general, it is concluded from the 3D surface graphs that the SR value tends to decrease at the middle values of AFR, SOD, and TS.

In the unconventional manufacturing method, the mixing ratio of pressurized water and abrasive particles is important. The required mixing ratio was sufficient for the material to be cut at the desired quality. While a poor mixing ratio affects the machining efficiency, a rich mixture results in the re-machining of the cut surfaces. In both cases, the quality of the machined surface was affected adversely. In the rich mixture, the chips removed from the workpiece surface by the abrasive particles were bombarded by new abrasive particles coming from behind before being removed by pressurized water. It even causes an unnecessary overlap of abrasive particles in the cutting zone. This situation reduces the cutting force of the abrasive particles that remove the chips from the workpiece. Overlapping abrasive particles create an impact cutting mechanism in the cutting zone.

When the effect of the SOD distance was analyzed, it was observed that as the nozzle moved away from the workpiece surface, the efficiency of the cutting process decreased, resulting in energy loss. It has been reported that the increase in SOD value leads to the spread of the water jet and loss of energy^25^. When cutting with AWJM, the jet gains cutting consistency with the aid of a focusing tube. After exiting the nozzle of the focusing tube, the particles were dispersed as a spray depending on the distance. The diameter of this spray increased as the distance increased, and the jet lost its consistency. When the distance is low, the water jet moves parallel to the cut surface of the part, and the abrasive particles in the jet move parallel to the cut surface of the part. Thus, micro-level chips were removed from the peaks of the cut surface. New abrasive particles coming from behind remove chips from the surface machined by the previous particle. Increasing the SOD caused the abrasive jet to lose coherence, disperse into a spray, and strike the cutting surface at oblique angles, resulting in particle embedding. The new abrasive particles coming from behind use some of their kinetic energy to remove the chips, whereas the rest of their energy is used to remove abrasive particles embedded in the material’s surface. This process causes loss of jet energy.

The TS, which depends on the material type and thickness, significantly affects the penetration of the jet into the machining depth to cut the workpiece. To ensure that the water jet cuts the workpiece with high quality, it is important to determine the TS. An indication of excessive TS is scratching on the lower part of the cutting surface. It has been reported that with a decrease in cutting speed, the SR value decreases, and the width of the primary zone without machining marks increases^8^. It is known that when turning the workpiece in conventional manufacturing, marks similar to screw marks are formed on the spindle surface with an increase in the feed rate. In AWJM, an increase in the TS results in marks on the machined surface similar to those in turning. In addition, because the penetration time of the water jet into the cut surface of the workpiece decreases with an increase in TS, the grinding effect of the water jet al.so decreases. This negatively affects the quality of the produced surface.

**Fig. 5:**
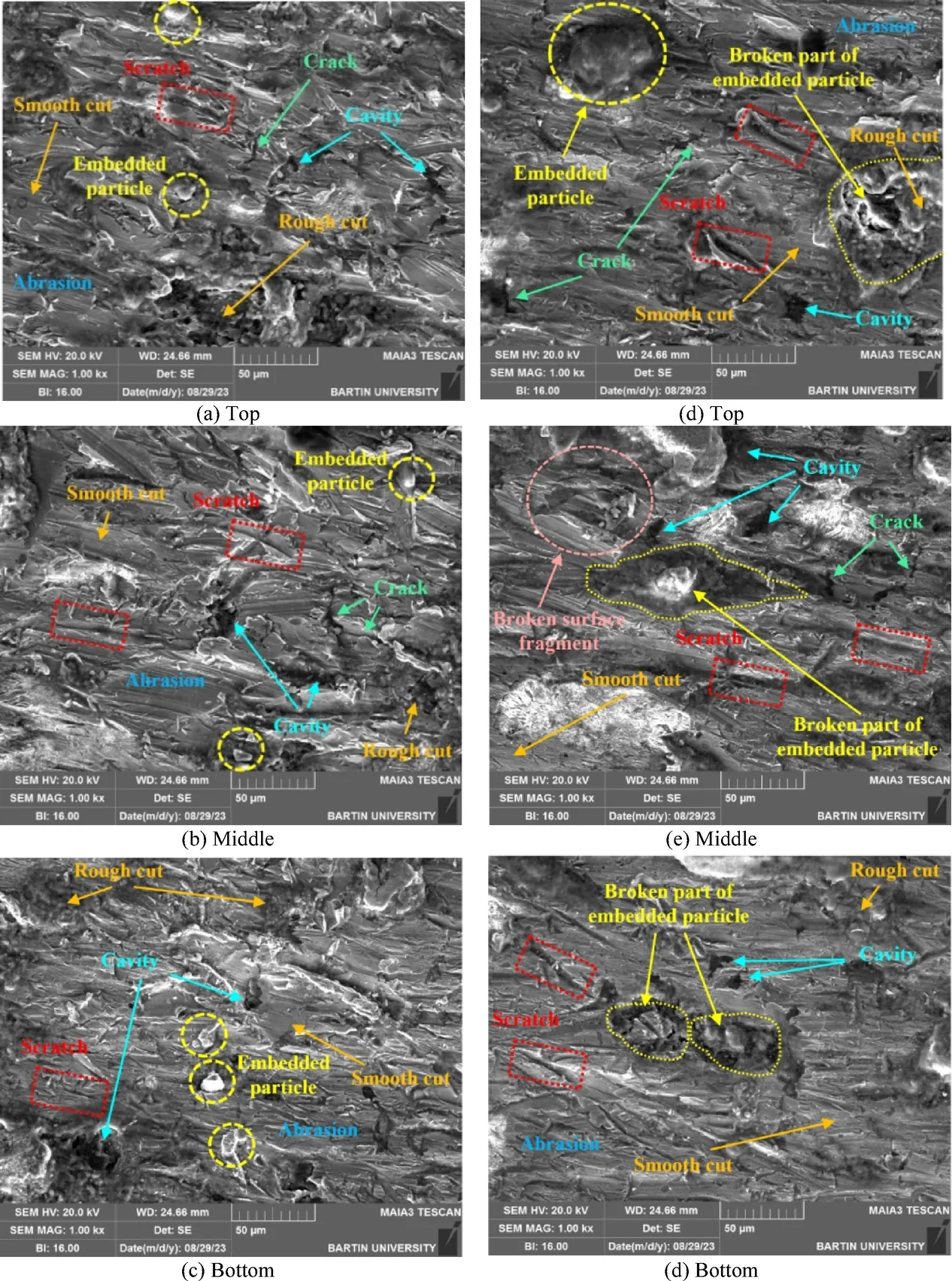
SEM images of AWJM for best surface (**(a)**, **(b)**, **(c)**) and worst surface (**(d)**, **(e)**, **(f)**).

The SEM images of the machining of CGI materials with the AWJM are shown in Fig. 5. Examination of SEM images revealed that machining parameters in AWJM influence the metal removal mechanics, confirming the coexistence of abrasive wear and impact erosion mechanisms during the processing of CGI. The presence of micro-grooves and scratches indicates abrasive wear, which occurs when abrasive particles act as cutting tools and sustain tangential movements along the surface. In contrast, crater-like cavities and micro-cracks reveal impact erosion, arising from high-velocity particle collisions that generate localized depressions, material fragmentation, and brittle fracture zones. Together, these features provide clear evidence that both mechanisms operate simultaneously during the process. A part of the abrasive particle is first embedded on the surface of the material to be machined owing to the pressure of the water jet. The depth of the embedded abrasive particle depends on the hardness ratio of the workpiece and abrasive particle. Then, the remaining part of the abrasive particle moves forward on the surface where it is embedded under the effect of the pressure and removes chips at the micro-level. This situation is described by the abrasive wear mechanism. With the grinding effect of abrasive particles with high kinetic energy, smoother surfaces are formed, which is a desired outcome. The SEM images show the particles embedded in the surface and scratches on the surface of the samples. It can be said that the scratches in the upper cutting zone caused by the kinetic energy of the water jet are deeper than the scratches in the lower cutting zone. This can be explained by the decrease in the kinetic energy of the water jet in the lower cutting zone of the workpiece. A similar effect has been observed in previous studies^5,7,8,29^. The SR values increased from the upper to the lower parts. Another issue is the surface cracks and cavities caused by the impact of abrasive particles on the surface owing to pressure. When the SEM images were examined, it was observed that the surface cracks present in the upper and middle cutting zones were absent in the lower cutting zone. Similarly, the depth of the cavities formed on the surface decreased in the lower region. This can be explained by the loss of kinetic energy of the water jet in the lower-cutting zone. Surface cracks and cavities caused by the impact of abrasive particles on the workpiece surface negatively affect the surface quality and create rough-cut surfaces. Surface cracks and cavities observed in the SEM images were directly associated with AFR and TS parameters. AFR determines the particle density and impact frequency per unit surface area, whereas TS controls the jet–surface interaction time and energy concentration. Higher AFR increases both the frequency and energy of particle collisions, thereby intensifying the distribution of concentrated impact energy and leading to more pronounced embedding marks, micro-cracks, and localized fracture zones. Conversely, lower TS results in prolonged jet exposure on the same region, producing wider cavities and deeper cracks. Collectively, these two parameters quantitatively govern the size and severity of cracks and cavities; notably, the combination of high AFR and low TS maximizes damage intensity by amplifying both abrasive wear and impact erosion mechanisms. Figure 5 (d-f) shows particles embedded in the surface but broken by the abrasive particle coming from behind, owing to the increase in the AFR. In this case, the abrasive wear mechanism started but could not be completed because the abrasive particles embedded in the workpiece were fractured. The roughness of the surface where the broken abrasive particles were embedded increased. Therefore, the surface quality decreased. Figure 5 (e) shows that with the impact cutting mechanism, the abrasive particle strikes the workpiece and causes the workpiece surface to fracture. This situation is even more serious with brittle and fragile materials. When the material is ductile, an initial surface crack forms when an abrasive particle strikes the cutting surface. It is crushed by the impact of particles coming from behind and breaks away from the surface as microchips.

The SEM images revealed distinct microstructural differences across the upper, middle, and lower regions during the AWJM of CGI, which directly correlated with variations in SR. In the upper region, the jet was the most stable and focused at the moment of impact, allowing abrasive particles to strike the surface with maximum kinetic energy. This produced uniform erosion and consequently lower SR values. In the middle region, increased turbulence and particle scattering caused partial energy dispersion, reducing cutting uniformity and resulting in moderate SR values. In the lower region, the jet lost coherence, and abrasive particles impacted the surface at random angles. This leads to less controlled material removal, producing rougher surfaces and larger taper angles. Collectively, these observations confirm that the microstructural features identified in SEM images are directly linked to the progressive changes in SR across different cutting depths.

### Kerf width

The arithmetic means of the S/N ratios of each level, differences, and degree of influence of the AWJM parameters on KW are presented in Table 10. Accordingly, when the control factors affecting KW were ranked according to their degree of influence, TS ranked 1st, AFR ranked 2nd, and SOD ranked 3rd. The optimum machining parameters for KW were the levels at which each factor had the highest S/N ratio. The largest S/N ratio of each factor (marked with *) is shown in Table 10. Thus, the optimum conditions for KW were determined. The optimal parameter combination for minimizing the KW was an AFR of 244 g/min, SOD of 1.5 mm, and TS of 25 mm/min.

**Table 10:** Average S/N ratios of AWJM parameters for the KW.

Levels	A	B	C
1	3.021*	2.907*	2.260
2	2.603	2.668	2.627
3	2.321	2.371	3.058*
Difference	0.700	0.536	0.797
Rank	2	3	1

* = Optimal Level.

**Fig. 6:**
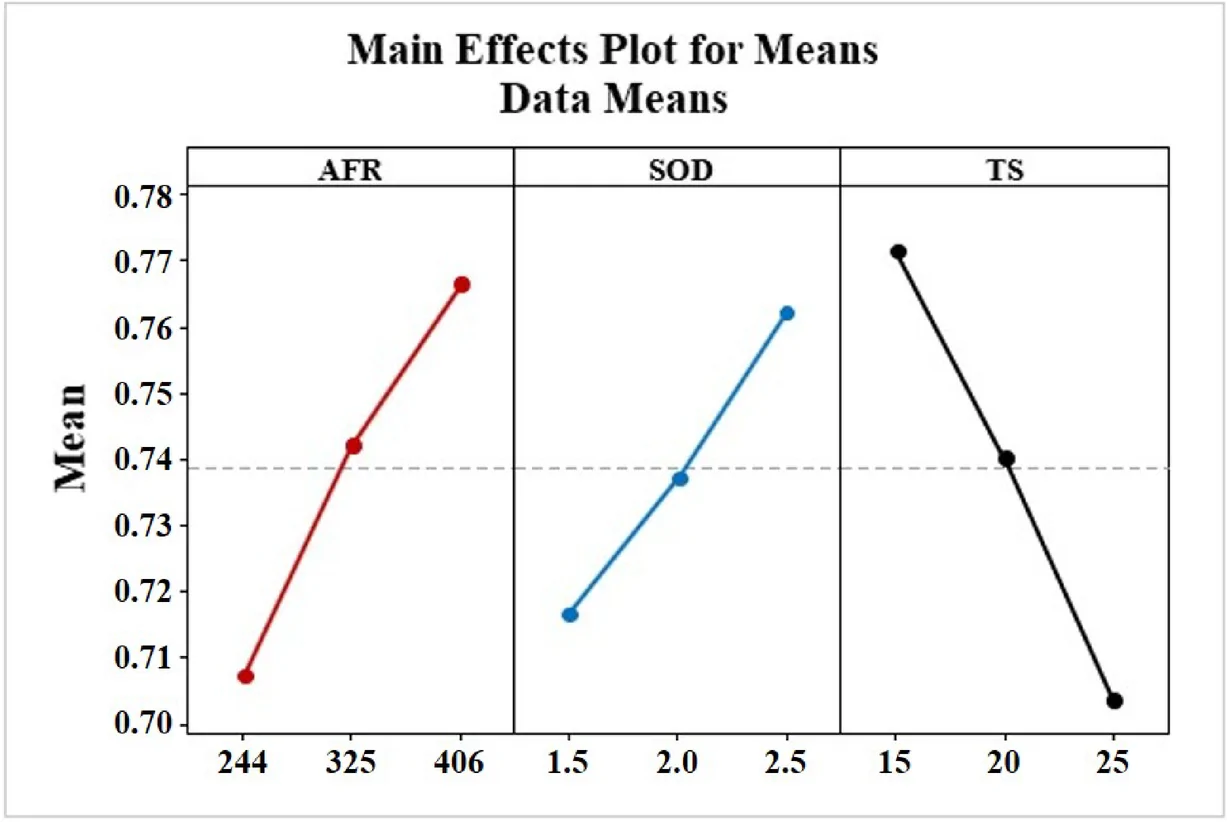
Main effect of AWJM parameters for KW.

The effect graphs of each factor on KW according to the average S/N ratio are shown in Fig. 5. When the graph is examined, it is observed that the KW value increases while the increase in both AFR and SOD, and decreases with the increase in TS. Similarly, it was found that the upper KW decreased with an increase in TS and a decrease in AFR^23^. In this study, it is clear from the graph in Fig. 6 that KW decreases with an increase in TS and a decrease in AFR. The MRR reportedly increases with an increase in the AFR^19^. This is an expected outcome. As the abrasive density in the pressurized water increased, the grinding effect of the water jet increased. In parallel with the increase in MRR, KW also increased. With the decrease in TS, the penetration time of the water jet per unit area increased, resulting in an increase in the KW. As the diameter of the jet exiting the nozzle increased with increasing SOD, a larger surface area of the workpiece was in contact with the water jet.

ANOVA was carried out at a 5% significance level (95% confidence level) to determine the effects and significance of AWJM parameters and their interactions on KW, with the results presented in Table 11. According to the ANOVA results, the most important factor affecting KW was TS (39.35%). Other factors affecting KW were AFR (30.46%) and SOD (17.80%), respectively. In terms of the interaction of factors, there was an interaction of AFR and TS with 5.44%, while the effects of other interactions were statistically insignificant (F-test < F-table). Therefore, the interactions between AFR and SOD, and between SOD and TS were statistically negligible.

**Table 11:** ANOVA result for the kerf width (KW).

Factors	DF	SS	V	F-test	PD
AFR (A)	2	0.0162	0.0081	56.67	30.458
SOD (B)	2	0.0095	0.0047	33.12	17.803
TS (C)	2	0.021	0.0105	73.20	39.346
A$\:\times\:$B	4	0.0012	0.0003	2.15	2.307
A$\:\times\:$C	4	0.0029	0.0007	5.06	5.441
B$\:\times\:$C	4	0.0013	0.0003	2.32	2.495
Error	8	0.0011	0.0001		2.150
Total	26	0.0532	0.02470		

Model summary *S* = 0,011954; $$\:{R}^{2}$$=97.85%; $$\:{R}_{adj}^{2}$$=93.01%.

DF: Degree of Freedom, SS: Sum of Squares, V: Variance, PD: Percentage of.

Distribution F-Table (0.05;2;8) = 4.46, F-Table (0.05;4;8) = 3.84.

### Kerf taper angle

The arithmetic means of the S/N ratios of each level, differences, and degree of influence of the AWJM parameters on KTA are presented in Table 12. Accordingly, when the control factors affecting KTA were ranked according to their degree of influence, AFR ranked 1st, SOD ranked 2nd, and TS ranked 3rd. The optimum machining parameters for KTA were the levels at which each factor had the highest S/N ratio. The largest S/N ratio of each factor (marked with *) is shown in Table 12. Thus, the optimum conditions for KTA were determined. The optimal parameter combination for minimizing the KTA was an AFR of 325 g/min, SOD of 2.5 mm, and a TS of 15 mm/min.

**Table 12:** Average S/N ratios of AWJM parameters for the kerf taper angle (KTA).

Levels	A	B	C
1	19.64	17.37	20.33*
2	25.88*	18.56	17.82
3	10.51	20.11*	17.88
Difference	15.37	2.74	2.51
Rank	1	2	3

* = Optimal Level.

**Fig. 7:**
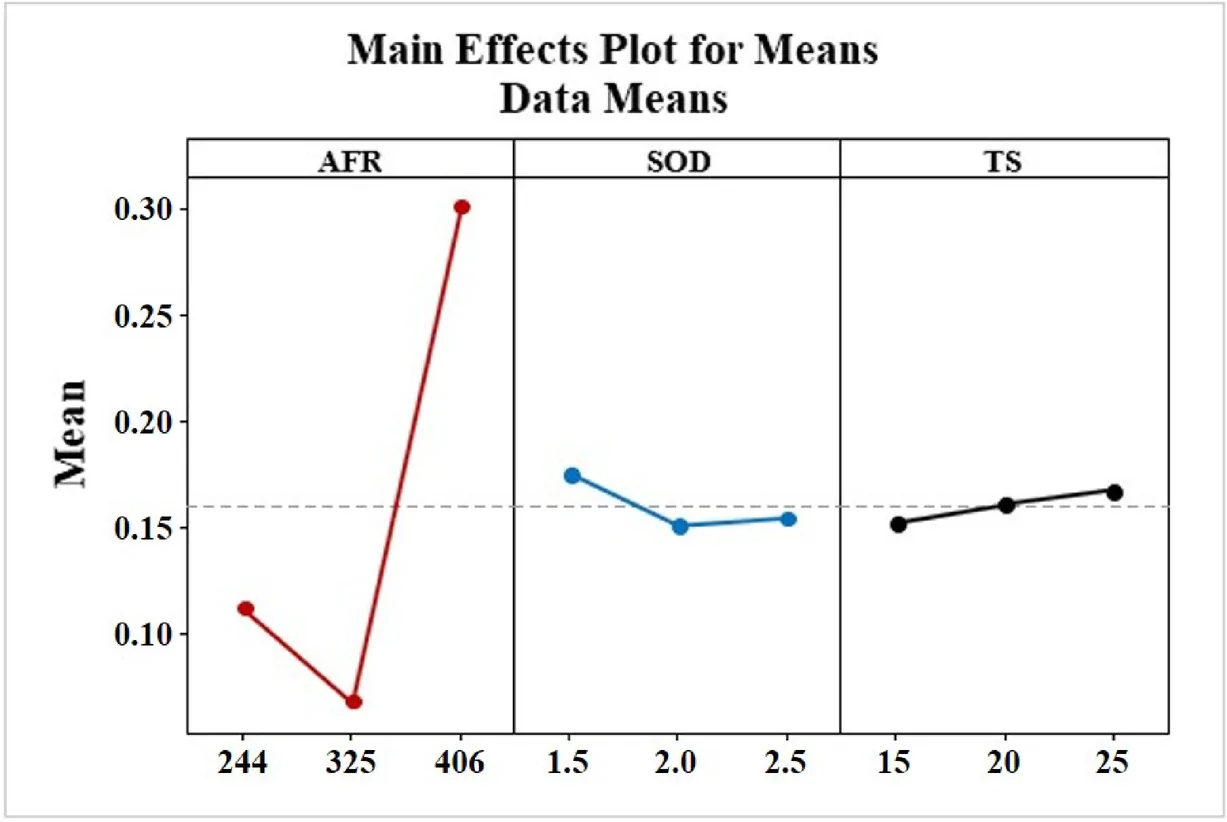
Main effect of AWJM parameters for KTA.

The effect graphs of each factor on KTA according to the average S/N ratio are shown in Fig. 7. When the graph is examined, it is observed that the KTA decreases while the AFR increases from 244 to 325 g/min, and the KTA value increases while the AFR increases from 325 to 406 g/min. The KTA value decreased while the SOD distance increased from 1.5 mm to 2 mm, and the KTA value increased while increasing from 2 mm to 2.5 mm. The KTA value increased with increasing TS value. As TS increased, KTA increased as the penetration time of the water jet to a depth of 10 mm decreased. In a previous study, it was concluded that an increase in SOD and TS levels increased KTA^24^.

ANOVA was carried out at a 5% significance level (95% confidence level) to determine the effects and significance of AWJM parameters and their interactions on KTA, with the results presented in Table 13. According to the ANOVA results, the most important factor affecting the KTA was AFR (86.52%). However, the effects of factors other than AFR and their interactions on KTA were statistically insignificant (F-test < F-table). Therefore, the effect of AWJM parameters and their interactions other than AFR is statistically negligible.

**Table 13:** ANOVA result for the kerf taper angle (KTA).

Factors	DF	SS	V	F-test	PD
AFR (A)	2	0.2756	0.1378	54.363	86.52
SOD (B)	2	0.0032	0.0016	0.624	0.99
TS (C)	2	0.0010	0.0005	0.199	0.32
A$\:\times\:$B	4	0.0061	0.0015	0.598	1.90
A$\:\times\:$C	4	0.0032	0.0008	0.319	1.02
B$\:\times\:$C	4	0.0092	0.0023	0.905	2.88
Error	8	0.0203	0.0025		6.37
Total	26	0.3186	0.01470		

Model summary *S* = 0,0503491; $$\:{R}^{2}$$=93.63%; $$\:{R}_{adj}^{2}$$=79.31%.

DF: Degree of Freedom, SS: Sum of Squares, V: Variance, PD: Percentage of.

Distribution F-Table (0.05;2;8) = 4.46, F-Table (0.05;4;8) = 3.84.

### Process–response interactions

According to the ANOVA results, AFR regulates the erosive capacity and stability of the jet, which in turn directly influences both SR and KTA. Deviations in AFR led to irregular cutting and micro-grooving, thereby increasing SR values. From the perspective of KTA, insufficient AFR reduces energy transmission along the depth, resulting in a widening of the taper angle. In contrast to SR and KTA, which are governed by abrasive particle density and energy distribution, KW is more sensitive to the duration of jet–material interaction at each cutting location. Fundamentally, KW represents a function of the MRR per unit length, which is controlled by the TS. These findings are consistent with the data obtained from experimental studies.

The optimized parameter combination (AFR = 325 g/min, SOD = 1.5 mm, and TS = 20 mm/min) established a balance between jet stability and erosion efficiency. At an AFR of 325 g/min, the jet carried a sufficient number of abrasive particles to sustain a dense and consistent flow, whereas an SOD of 1.5 mm ensured that the jet remained focused and stable, enabling precise energy transfer to the workpiece. A TS of 20 mm/min further balanced efficiency with controlled erosion, thereby producing narrow kerfs and smooth surfaces. Collectively, these parameters yielded smooth surfaces, narrow kerf widths, and low taper angles without compromising the cutting speed or precision, confirming the balance achieved between stability and efficiency.

## Conclusion

In this study, the influence of key process parameters, namely, AFR, SOD, and TS, on output responses, including SR, KW, and KTA, during the AWJM of CGI was investigated. The Taguchi L27 design and ANOVA were used to identify the dominant parameters and optimize the process conditions. CGI materials, which are difficult to machine using conventional methods, were successfully machined using AWJM, and the following major results were obtained:

- **Influence of process parameters**: AFR was the most significant factor affecting SR and KTA, whereas TS predominantly influenced KW. SOD exhibited only a minor contribution to all the response variables. The effect of the interactions of the machining parameters was insignificant based on the F-test results. ANOVA confirmed that AFR contributed 51.46% to the variation in SR and 86.52% to the variation in KTA, whereas TS contributed 39.35% to the variation in KW.
- **Optimized machining conditions**: The optimum parameter combinations were determined as follows — for minimum SR: **325 g/min AFR**,** 1.5 mm SOD**,** and 20 mm/min TS**; for minimum KW: **244 g/min AFR**,** 1.5 mm SOD**,** and 25 mm/min TS**; and for minimum KTA: **325 g/min AFR**,** 2.5 mm SOD**,** and 15 mm/min TS.** Intermediate parameter levels generally produced the best surface quality and kerf geometry owing to the balanced interaction between the erosion energy and jet stability.
- **Mechanical insights**: SEM observations revealed that both abrasive wear and impact erosion mechanisms managed material removal in CGI. Excessive AFR or high SOD values cause abrasive interference, surface cracking, and jet defocusing, resulting in degraded surface integrity. Optimal parameter settings enhance jet stability, enabling the precise and thermally damage-free machining of CGI components.

## Data Availability

All data generated or analysed during this study are included in this published article.
